# Selenomethionine Attenuates Hydrogen Peroxide-Induced Oxidative Injury and Modulates Unfolded Protein Response-Related Markers in Murine C2C12 Myoblasts

**DOI:** 10.3390/antiox15091099

**Published:** 2026-08-31

**Authors:** Xin Xin, Xiangzi Li, Shiyu Jin, Xiaoxu Huang, Xu Gao, Qiang Li, Xin Jin

**Affiliations:** 1 Department of Veterinary Medicine, College of Agriculture, Yanbian University, Yanji 133002, China; 15948320553@163.com (X.X.); 17624252084@163.com (S.J.); m1198599120@163.com (X.H.); gaoxu@ybu.edu.cn (X.G.); 2 Engineering Research Center of North-East Cold Region Beef Cattle Science & Technology Innovation, Ministry of Education, College of Agriculture, Yanbian University, Yanji 133002, China; 0000003144@ybu.edu.cn; 3 Laboratory Animal Center, College of Agriculture, Yanbian University, Yanji 133002, China

**Keywords:** selenomethionine, oxidative stress, antioxidant defense, C2C12 myoblasts, unfolded protein response

## Abstract

Oxidative stress disrupts skeletal muscle cell survival and proteostasis, thereby compromising animal skeletal muscle health. As an organic selenium source, selenomethionine (SeMet) participates in antioxidant defense and selenoprotein regulation. However, its protective role in hydrogen peroxide (H_2_O_2_)-induced injury in C2C12 myoblasts and its association with unfolded protein response (UPR)-related molecular changes remain incompletely understood. This study investigated whether SeMet alleviates H_2_O_2_-induced injury in C2C12 myoblasts and whether this effect is associated with UPR-related marker changes. C2C12 myoblasts were treated with H_2_O_2_ and SeMet, and cell survival was evaluated using cell viability assays and calcein acetoxymethyl ester/propidium iodide co-staining. The expression of selenoprotein-, UPR-, and apoptosis-related genes was examined by quantitative real-time PCR, and corresponding protein levels were assessed by Western blotting. SeMet improved cell survival under oxidative stress and increased *Selenof* mRNA and SELENOF protein expression. It also attenuated aberrant changes in endoplasmic reticulum and mitochondrial UPR-related markers and reduced apoptosis-associated molecular responses. These findings indicate that SeMet protects C2C12 myoblasts against H_2_O_2_-induced oxidative injury. This protective effect may be associated with changes in SELENOF expression, UPR-related markers, and apoptosis-associated molecular responses, providing molecular evidence for its potential role in maintaining skeletal muscle cell homeostasis.

## 1. Introduction

Skeletal muscle satellite cells (SMSCs) are essential myogenic precursor cells that contribute to skeletal muscle regeneration after injury [1]. Under physiological conditions, these cells remain quiescent but can be activated upon tissue damage to initiate muscle repair [2]. External stimuli can induce reactive oxygen species (ROS) production in myogenic cells, while low physiological levels of ROS contribute to cellular adaptive responses and skeletal muscle regeneration [3]. However, persistent stress can lead to excessive ROS accumulation, disrupt cellular redox homeostasis, reduce cell viability, and ultimately induce apoptosis [4]. Thus, oxidative stress-induced myogenic cell dysfunction may limit skeletal muscle repair.

As oxidative stress progresses, excessive ROS can disrupt proteostasis by promoting oxidative protein damage and impairing protein folding within the endoplasmic reticulum (ER) and mitochondria [5,6]. The accumulation of unfolded or misfolded proteins subsequently activates unfolded protein response (UPR)-related pathways, including the ER-UPR and mitochondrial UPR (MT-UPR) [7,8]. ER-UPR primarily restores ER proteostasis through protein-folding and degradation pathways, whereas MT-UPR maintains mitochondrial proteostasis by coordinating chaperone- and protease-mediated mitochondrial quality control [7,8]. These pathways collectively form a cellular stress-adaptive network that sustains proteostasis and mitochondrial function in myogenic cells, whereas dysfunction of this network may compromise cellular homeostasis and regenerative capacity under oxidative stress [9,10,11,12].

Selenium is an essential trace element that exerts its physiological effects primarily through selenoproteins [13]. Selenium and selenoproteins are closely associated with skeletal muscle function, and selenium deficiency can lead to skeletal muscle dysfunction [14]. Several selenoproteins participate in redox regulation to alleviate oxidative damage and maintain cellular homeostasis [15,16]. Among selenium-containing proteins, SELENOF is involved in maintaining ER protein homeostasis, whereas SELENOP plays an important role in selenium transport and distribution [17,18]. These distinct functions suggest that selenoproteins may contribute to cellular adaptation to oxidative stress through different regulatory mechanisms. In a related in vivo study, Jing et al. showed that dietary hydroxy-selenomethionine alleviated oxidative stress-induced skeletal muscle growth inhibition in piglets by reducing ROS accumulation and regulating UPR-related pathways [19]. These findings provide an important mechanistic clue that selenium-mediated skeletal muscle protection may be closely linked to the maintenance of organelle proteostasis under oxidative stress.

However, the cellular basis of this protective process, particularly how selenomethionine (SeMet) affects ER-UPR- and MT-UPR-related responses in myogenic cells exposed to oxidative stress, remains unclear. Therefore, this study used hydrogen peroxide (H_2_O_2_)-exposed C2C12 myoblasts as an in vitro oxidative stress model to evaluate SeMet protection, focusing on ER-UPR- and MT-UPR-related responses.

## 2. Materials and Methods

### 2.1. Cell Culture and Experimental Treatment

C2C12 myoblasts were obtained from the Engineering Research Center of North-East Cold Region Beef Cattle Science and Technology Innovation, Ministry of Education, China. Cells were cultured in Dulbecco’s modified Eagle’s medium (DMEM; Gibco, Thermo Fisher Scientific, Suzhou, China) supplemented with 10% fetal bovine serum (FBS; Gibco, Thermo Fisher Scientific) and 1% penicillin–streptomycin (Beyotime, Shanghai, China) under standard conditions at 37 °C with 5% CO_2_. At approximately 60% confluence, cells were maintained in DMEM containing 2% FBS for 12 h as a short-term serum reduction step to minimize serum-derived growth stimulation and improve experimental consistency. This brief low-serum exposure was not intended to induce myogenic differentiation or establish selenium-depleted conditions. Cells were exposed to H_2_O_2_ (Sigma-Aldrich, St. Louis, MO, USA) alone or co-treated with H_2_O_2_ and SeMet (Shanghai Yuanye Bio-Technology Co., Ltd., Shanghai, China) for 24 h. Cells were divided into four groups: control (standard culture), H_2_O_2_ group (1000 μmol/L H_2_O_2_ treatment), and two SeMet treatment groups (100 nmol/L or 200 nmol/L SeMet in combination with 1000 μmol/L H_2_O_2_).

### 2.2. Oxidative Stress Model Construction and SeMet Concentration Screening

Cell viability was assessed using the Cell Counting Kit-8 (CCK-8; APExBIO, Shanghai, China) assay. C2C12 myoblasts were seeded in 96-well plates at 1 × 10^3^ cells/well and cultured overnight at 37 °C with 5% CO_2_. Cells were exposed to H_2_O_2_ at 500, 600, 700, 800, 900, and 1000 μmol/L or SeMet at 10, 20, 50, 100, and 200 nmol/L for 24 h. Absorbance was measured at 450 nm using a microplate reader (Bio-Rad Laboratories, Hercules, CA, USA).

### 2.3. Cell Viability Assay by Calcein-AM/PI Co-Staining

The protective effects of SeMet were evaluated using a live/dead cell staining kit (Beyotime). Cells were seeded in 6-well plates at a density of 2 × 10^4^ cells/well. After H_2_O_2_ and/or SeMet treatment, the cells were washed with PBS (Solarbio, Beijing, China), and 1 mL of calcein acetoxymethyl ester/propidium iodide (Calcein-AM/PI) working solution was added to each well. Cells were incubated at 37 °C in the dark for 30 min and fluorescence images were captured using a fluorescence microscope (Olympus, Tokyo, Japan).

### 2.4. Total RNA Extraction and qRT-PCR

Total RNA was extracted using TRIzol reagent (Thermo Fisher Scientific). Subsequently, 1 μg of total RNA was reverse-transcribed using a FastKing RT Kit (Tiangen Biotech Co., Ltd., Beijing, China). Quantitative real-time PCR (qRT-PCR) was performed using SuperReal PreMix Plus (Tiangen Biotech Co., Ltd.) according to the manufacturer’s instructions. Each reaction was performed in a total volume of 20 μL. The amplification program was as follows: initial denaturation at 95 °C for 15 min, followed by 40 cycles of denaturation at 95 °C for 10 s, annealing at 55 °C for 30 s, and extension at 72 °C for 32 s. The full names and primer sequences of the analyzed genes are listed in Table 1. Glyceraldehyde-3-phosphate dehydrogenase (*Gapdh*) was used as the reference gene, and relative mRNA expression was calculated using the 2^−ΔΔCt^ method.

### 2.5. Western Blot Analysis

Cells were lysed with radioimmunoprecipitation assay buffer containing 1% phenylmethylsulfonyl fluoride (Solarbio). Protein concentration was determined using a bicinchoninic acid protein assay kit (Thermo Fisher Scientific). In total, 20 μg of protein from each sample was loaded into each lane, separated on 12.5% sodium dodecyl sulfate–polyacrylamide gels, and transferred onto polyvinylidene difluoride membranes. After blocking with 5% bovine serum albumin for 1 h at room temperature, membranes were incubated overnight at 4 °C with primary antibodies against selenoprotein F (SELENOF), selenoprotein P (SELENOP), 78 kDa glucose-regulated protein (GRP78), X-box binding protein 1 (XBP1), caseinolytic mitochondrial matrix peptidase proteolytic subunit (CLPP), c-Jun N-terminal kinase 2 (JNK2), BCL2-associated X protein (BAX), B-cell lymphoma 2 (BCL-2), and β-actin, followed by incubation with secondary antibodies (1:1000; Beyotime) for 2 h at room temperature. Detailed information regarding primary antibodies, including manufacturer, catalog number, clone, host species, and dilution, is provided in Supplementary Table S1. Bands were detected using enhanced chemiluminescence (ECL; Beyotime) and quantified using ImageJ software (version 1.51j8; National Institutes of Health, Bethesda, MD, USA). β-actin was used as the loading control, and target proteins were normalized to β-actin.

### 2.6. Statistical Analysis

Data were analyzed using GraphPad Prism 10.4. One-way analysis of variance (ANOVA) followed by Tukey’s multiple comparisons test was used for multiple comparisons. Differences were considered significant at *p* < 0.05. All experiments were independently performed three times. Technical replicates within each independent experiment were averaged before statistical analysis, and data are presented as mean ± SD from three independent experiments (*n* = 3).

## 3. Results

### 3.1. SeMet Attenuated H_2_O_2_-Induced Injury in C2C12 Myoblasts

**Figure 1:**
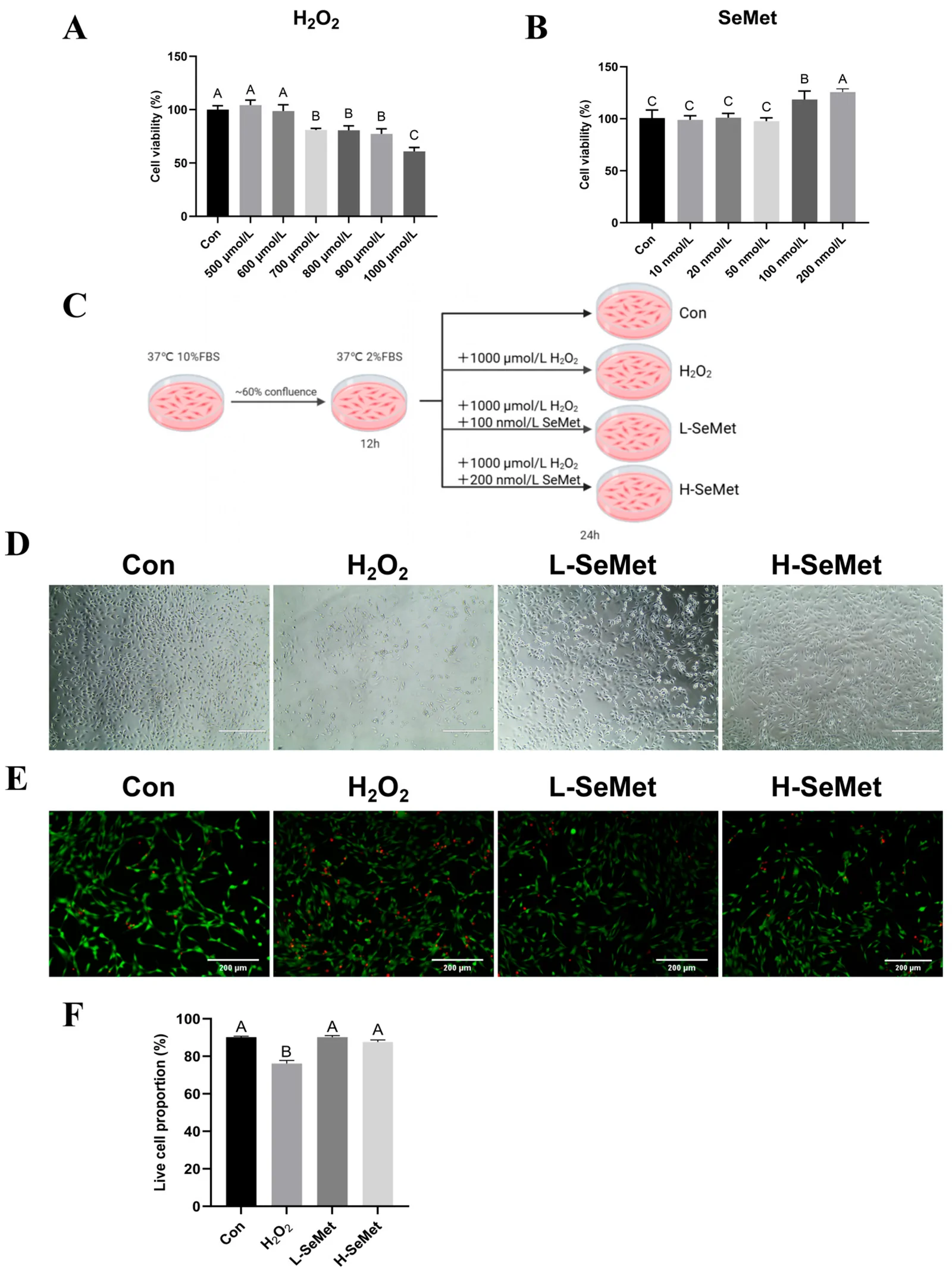
SeMet attenuated H_2_O_2_-induced oxidative injury in C2C12 myoblasts. (**A**) Effects of different H_2_O_2_ concentrations on C2C12 myoblast viability, as determined by the CCK-8 assay. (**B**) Effects of different SeMet concentrations on C2C12 myoblast viability in the absence of H_2_O_2_ treatment. (**C**) Schematic representation of the experimental groups: Con, untreated control; H_2_O_2_, 1000 μmol/L H_2_O_2_; L-SeMet, 100 nmol/L SeMet + 1000 μmol/L H_2_O_2_; H-SeMet, 200 nmol/L SeMet + 1000 μmol/L H_2_O_2_. (**D**) Representative bright-field images showing the morphology of C2C12 myoblasts after different treatments. Scale bar = 400 μm; magnification, ×100. (**E**) Representative fluorescence images of Calcein-AM/PI co-staining of C2C12 myoblasts. Live cells are shown in green, and dead cells are shown in red. Scale bar = 200 μm; magnification, ×100. (**F**) Quantitative analysis of live cell proportion based on Calcein-AM/PI co-staining. H_2_O_2_, hydrogen peroxide; SeMet, selenomethionine; CCK-8, Cell Counting Kit-8; Calcein-AM/PI, calcein acetoxymethyl ester/propidium iodide. Different letters above the bars indicate significant differences among groups (*p* < 0.05). Quantitative data are presented as mean ± SD from three independent experiments (*n* = 3).

To establish the oxidative stress model and determine appropriate SeMet concentrations, C2C12 myoblast viability was evaluated by CCK-8 assay. H_2_O_2_ reduced cell viability in a concentration-dependent manner, with 1000 μmol/L producing a marked inhibitory effect, whereas SeMet at 100 and 200 nmol/L significantly enhanced cell viability (Figure 1A,B). Accordingly, 1000 μmol/L H_2_O_2_ was selected for model induction, and 100 and 200 nmol/L SeMet were used as the low- and high-concentration treatments, respectively (Figure 1C).

Compared with the control group, H_2_O_2_ treatment markedly reduced cell density, induced cell shrinkage, and increased PI-positive dead cells. In contrast, SeMet treatment partially restored cell morphology and increased the proportion of viable cells under H_2_O_2_-induced oxidative stress, as confirmed by Calcein-AM/PI staining and quantitative analysis (Figure 1D–F).

### 3.2. SeMet Modulated SELENOF and SELENOP Expression in C2C12 Myoblasts

**Figure 2:**
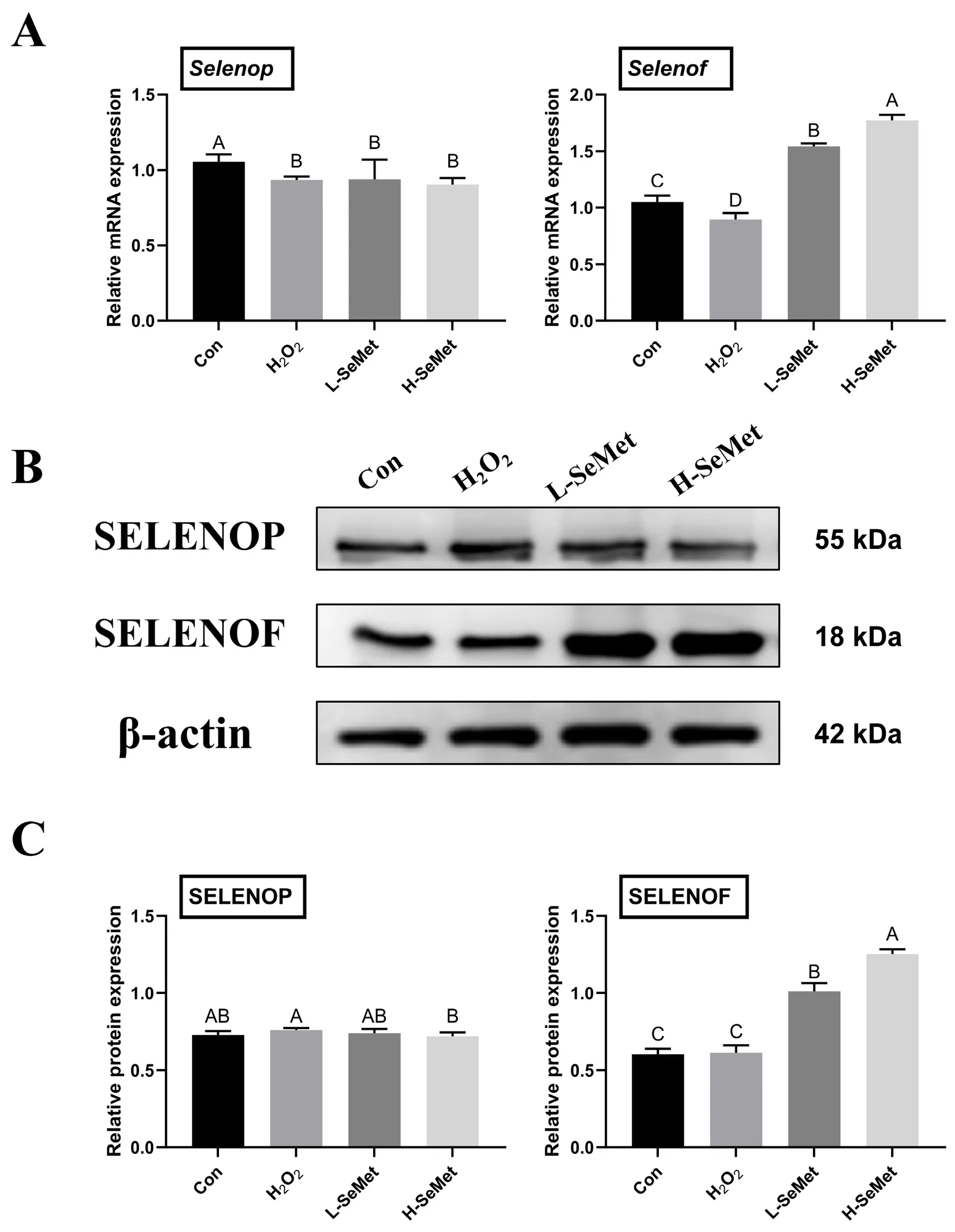
SeMet modulated SELENOF and SELENOP expression in C2C12 myoblasts under H_2_O_2_-induced oxidative stress. (**A**) Relative mRNA expression levels of *Selenop* and *Selenof* after different treatments, as determined by qRT-PCR. (**B**) Representative Western blot bands showing the protein expression of SELENOP and SELENOF. β-actin was used as the loading control. (**C**) Quantitative analysis of SELENOP and SELENOF protein expression normalized to β-actin. Con, untreated control; H_2_O_2_, 1000 μmol/L H_2_O_2_; L-SeMet, 100 nmol/L SeMet + 1000 μmol/L H_2_O_2_; H-SeMet, 200 nmol/L SeMet + 1000 μmol/L H_2_O_2_. H_2_O_2_, hydrogen peroxide; SeMet, selenomethionine; qRT-PCR, quantitative real-time PCR. Different letters above the bars indicate significant differences among groups (*p* < 0.05). Quantitative data are presented as mean ± SD from three independent experiments (*n* = 3).

To determine whether SeMet affected representative selenoproteins, *Selenof/Selenop* mRNA expression and SELENOF/SELENOP protein levels were measured. H_2_O_2_ treatment decreased *Selenop* mRNA expression, and SeMet did not restore its expression under oxidative stress (Figure 2A). *Selenof* mRNA expression was also reduced by H_2_O_2_, whereas SeMet markedly increased its expression in a concentration-dependent manner (Figure 2A). At the protein level, SELENOP showed limited changes among groups, while SeMet significantly increased SELENOF expression in H_2_O_2_-treated C2C12 myoblasts (Figure 2B,C).

### 3.3. SeMet Attenuated H_2_O_2_-Induced ER-UPR-Related Responses in C2C12 Myoblasts

**Figure 3:**
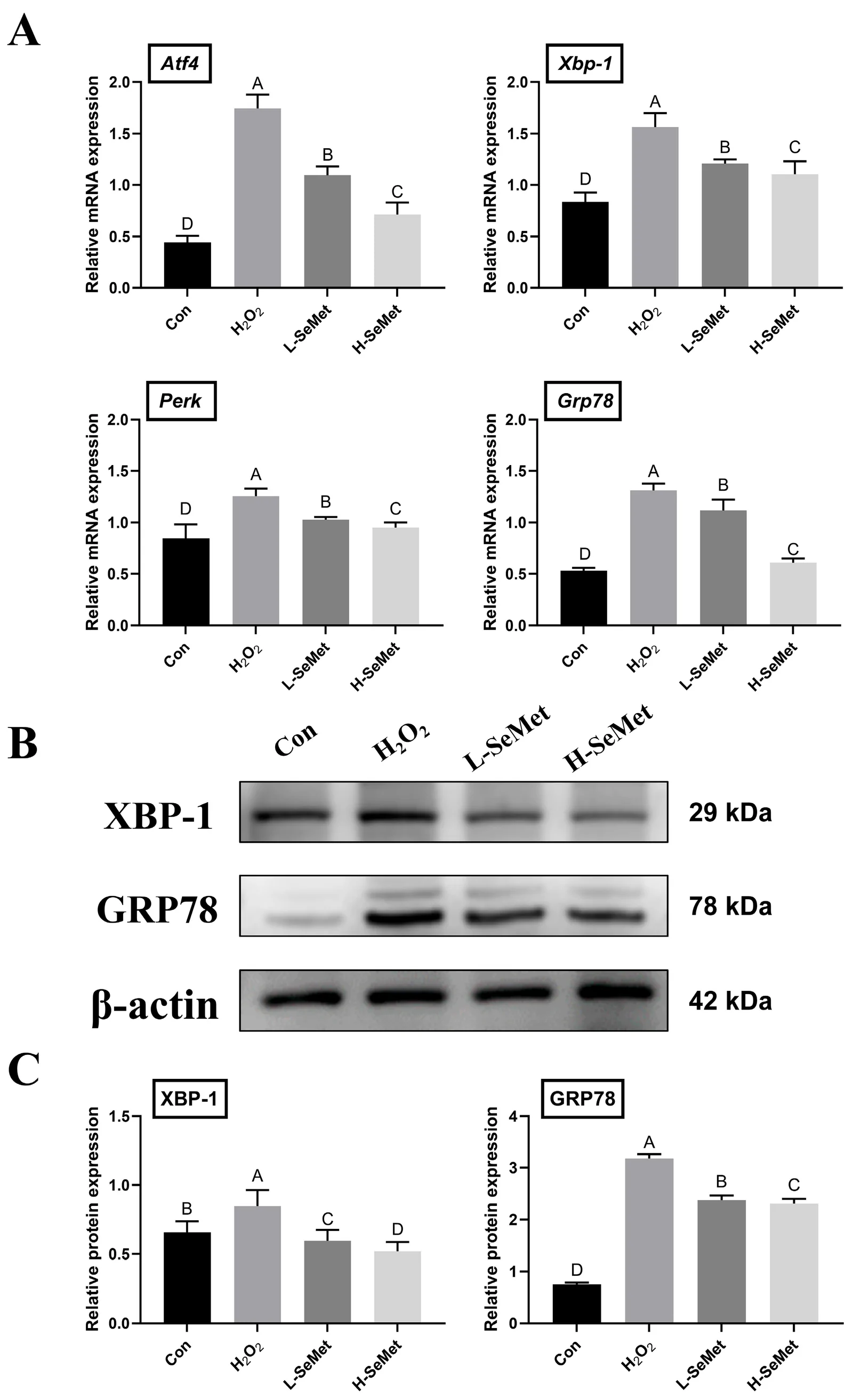
Effects of SeMet on ER-UPR-related markers in H_2_O_2_-treated C2C12 myoblasts. (**A**) Relative mRNA expression levels of *Atf4*, *Xbp1*, *Perk*, and *Grp78* after different treatments, as determined by qRT-PCR. (**B**) Representative Western blot bands showing XBP1 and GRP78 protein expression. β-actin was used as the loading control. (**C**) Quantitative analysis of XBP1 and GRP78 protein expression normalized to β-actin. Con, untreated control; H_2_O_2_, 1000 μmol/L H_2_O_2_; L-SeMet, 100 nmol/L SeMet + 1000 μmol/L H_2_O_2_; H-SeMet, 200 nmol/L SeMet + 1000 μmol/L H_2_O_2_. ER-UPR, endoplasmic reticulum unfolded protein response; H_2_O_2_, hydrogen peroxide; SeMet, selenomethionine; qRT-PCR, quantitative real-time PCR. Different letters above the bars indicate significant differences among groups (*p* < 0.05). Quantitative data are presented as mean ± SD from three independent experiments (*n* = 3).

H_2_O_2_ treatment markedly increased the mRNA expression of ER-UPR-related genes, including *Atf4*, *Xbp1*, *Perk*, and *Grp78*, whereas SeMet treatment significantly reduced their expression (Figure 3A). Consistently, at the protein level, H_2_O_2_ increased XBP1 and GRP78 expression, while SeMet treatment reduced both proteins in a concentration-dependent manner (Figure 3B,C). These results showed that SeMet attenuated H_2_O_2_-induced ER-UPR-related responses in C2C12 myoblasts.

### 3.4. SeMet Attenuated H_2_O_2_-Induced MT-UPR Related Responses in C2C12 Myoblasts

**Figure 4:**
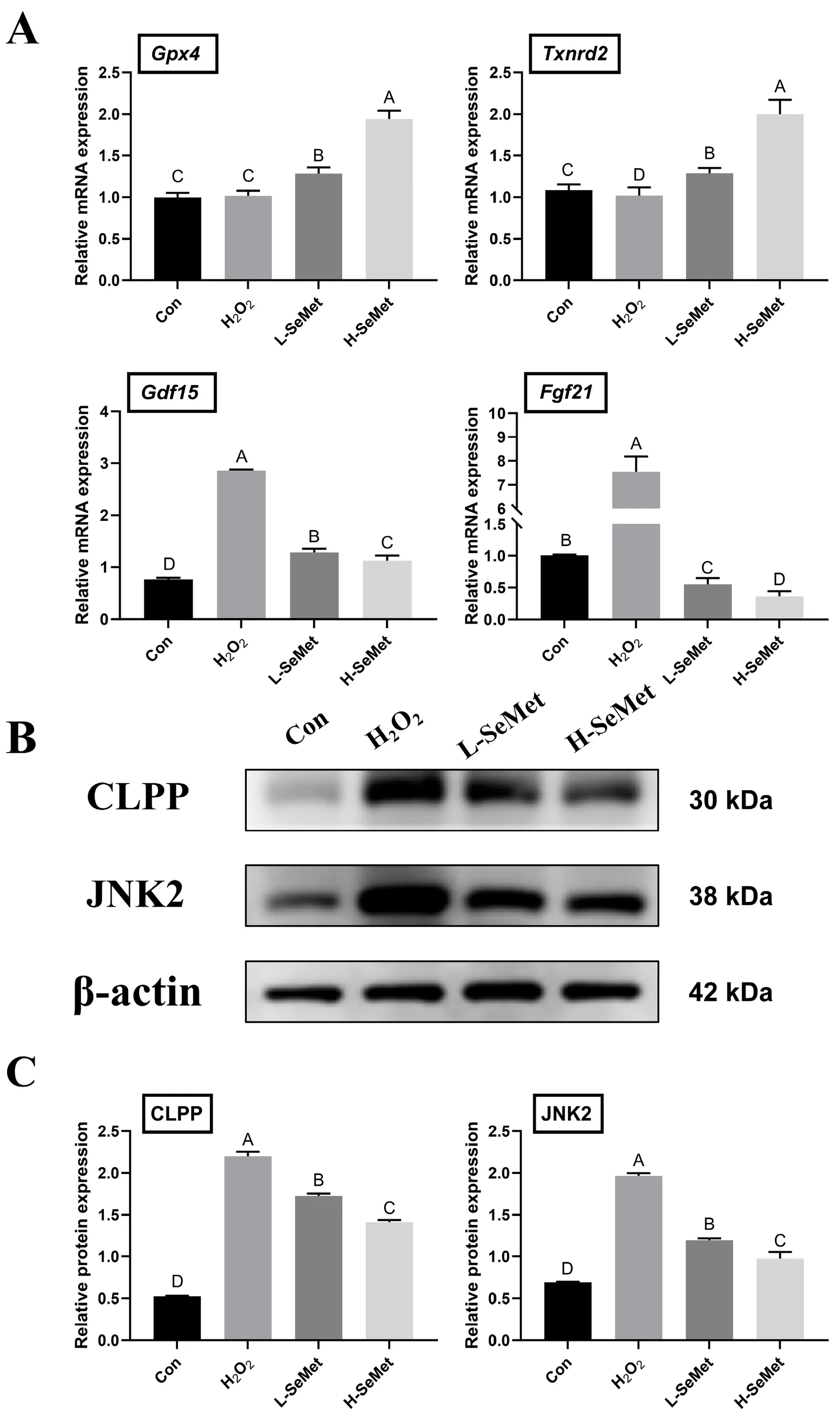
Effects of SeMet on MT-UPR-related markers in H_2_O_2_-treated C2C12 myoblasts. (**A**) mRNA expression levels of mitochondrial selenoprotein genes, including *Gpx4* and *Txnrd2*, and mitochondrial stress-related genes, including *Gdf15* and *Fgf21*, after different treatments, as determined by qRT-PCR. (**B**) Representative Western blot bands showing the protein expression of CLPP and JNK2. β-actin was used as the loading control. (**C**) Quantitative analysis of CLPP and JNK2 protein expression normalized to β-actin. Con, untreated control; H_2_O_2_, 1000 μmol/L H_2_O_2_; L-SeMet, 100 nmol/L SeMet + 1000 μmol/L H_2_O_2_; H-SeMet, 200 nmol/L SeMet + 1000 μmol/L H_2_O_2_. H_2_O_2_, hydrogen peroxide; SeMet, selenomethionine; MT-UPR, mitochondrial unfolded protein response; qRT-PCR, quantitative real-time PCR. Different letters above the bars indicate significant differences among groups (*p* < 0.05). Quantitative data are presented as mean ± SD from three independent experiments (*n* = 3).

To determine whether SeMet affected MT-UPR-related responses under oxidative stress, the expression of mitochondrial redox- and stress-related markers was examined. SeMet treatment increased the mRNA expression of the redox-related genes *Gpx4* and *Txnrd2* in a concentration-dependent manner (Figure 4A). In contrast, H_2_O_2_ treatment markedly upregulated the mitochondrial stress-related genes *Gdf15* and *Fgf21*, whereas SeMet treatment reduced their expression in H_2_O_2_-exposed cells (Figure 4A). At the protein level, H_2_O_2_ increased CLPP and JNK2 expression, whereas SeMet treatment decreased both proteins, with a stronger effect observed in the high-concentration group (Figure 4B,C).

### 3.5. SeMet Modulated Apoptosis-Related Markers in H_2_O_2_-Exposed C2C12 Myoblasts

**Figure 5:**
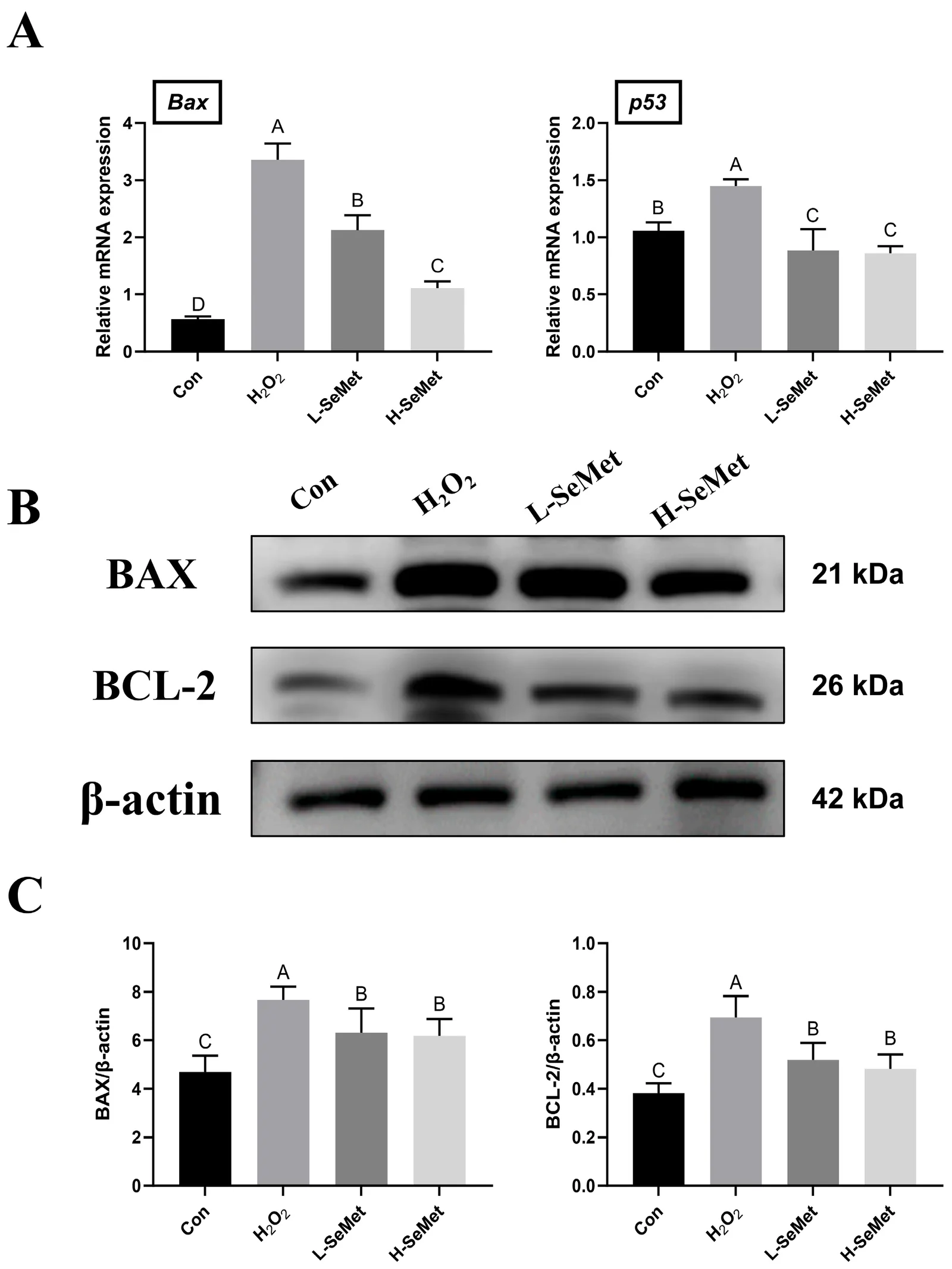
Effects of SeMet on apoptosis-related markers in H_2_O_2_-treated C2C12 myoblasts. (**A**) Relative mRNA expression levels of *Bax* and *p53* after different treatments, as determined by qRT-PCR. (**B**) Representative Western blot bands showing BAX and BCL-2 protein expression. β-actin was used as the loading control. (**C**) Quantitative analysis of BAX and BCL-2 protein expression normalized to β-actin. Con, untreated control; H_2_O_2_, 1000 μmol/L H_2_O_2_; L-SeMet, 100 nmol/L SeMet + 1000 μmol/L H_2_O_2_; H-SeMet, 200 nmol/L SeMet + 1000 μmol/L H_2_O_2_. H_2_O_2_, hydrogen peroxide; SeMet, selenomethionine; qRT-PCR, quantitative real-time PCR. Different letters above the bars indicate significant differences among groups (*p* < 0.05). Quantitative data are presented as mean ± SD from three independent experiments (*n* = 3).

H_2_O_2_ treatment markedly increased the mRNA expression of *Bax* and *p53*, whereas SeMet treatment reduced their expression in H_2_O_2_-exposed C2C12 myoblasts (Figure 5A). At the protein level, H_2_O_2_ increased the expression of BAX and BCL-2, while SeMet treatment decreased both proteins compared with the H_2_O_2_ group (Figure 5B,C).

## 4. Discussion

Oxidative stress can inhibit skeletal muscle regeneration by impairing the function of SMSCs [20]. Therefore, maintaining redox balance and organelle homeostasis is essential for preserving myoblast viability under stress conditions. In the present study, SeMet treatment alleviated H_2_O_2_-induced morphological damage and improved cell viability in C2C12 myoblasts, preliminarily confirming its protective effect against oxidative stress injury. These results provide a rationale for further investigating whether SeMet-mediated protection is associated with selenoprotein expression and organelle stress responses.

In this study, 1000 μmol/L H_2_O_2_ was selected to establish the oxidative stress model, which is comparable to the H_2_O_2_ concentrations commonly used to induce cytotoxicity in C2C12 cells [21]. SeMet at 100 and 200 nmol/L improved cell viability and partially restored cell morphology under H_2_O_2_ treatment (Figure 1B,D). Notably, the SeMet concentrations used in the present study were lower than the 500 nmol/L SeMet applied in previous C2C12 studies [22], suggesting that relatively low-concentration SeMet was sufficient to exert a protective effect in the present oxidative stress model. From a nutritional perspective, SeMet is an organic selenium source that can be nonspecifically incorporated into body proteins in place of methionine, thereby forming a mobilizable selenium reserve [23,24]. It can also be metabolized to provide selenium for selenocysteine and selenoprotein biosynthesis [25,26].

This nutritional property provides a rationale for examining whether SeMet-mediated protection in C2C12 myoblasts is associated with selenoprotein responses under oxidative stress. Based on the focus of this study, SELENOF and SELENOP were selected as representative selenoproteins with distinct biological relevance: SELENOF is associated with ER redox regulation, protein folding, and UPR homeostasis [17], whereas SELENOP mainly participates in selenium transport and systemic selenium homeostasis [18]. In this study, SeMet increased *Selenof* mRNA and SELENOF protein expression, while SELENOP showed a relatively modest response (Figure 2A–C). This SELENOF pattern differs from previous pig liver findings, in which dietary oxidative stress tended to increase SELENOF protein abundance and OH-SeMet supplementation reduced it while alleviating ER stress [27]. Together with related porcine skeletal muscle findings showing non-linear SELENOF responses to OH-SeMet [19], these results suggest that SELENOF regulation may vary according to selenium source, tissue type, and oxidative stress model.

Oxidative stress can promote ER-UPR-related pathways [6]. GRP78, PERK/ATF4, and XBP1 are representative ER stress-related UPR markers that indicate increased protein-folding demand and activation of adaptive ER stress signaling under stress conditions [28]. In the present study, H_2_O_2_ increased the expression of *Atf4*, *Xbp1*, *Perk*, and *Grp78* (Figure 3A), together with elevated XBP1 and GRP78 protein levels (Figure 3B,C), whereas SeMet reduced these changes, especially at the high concentration. These results showed that SeMet may alleviate H_2_O_2_-induced ER stress burden and help maintain ER protein homeostasis in C2C12 myoblasts. ER stress signaling may influence mitochondrial homeostasis [29]. Therefore, the effects of SeMet on mitochondrial stress- and MT-UPR-related markers were further examined in H_2_O_2_-treated C2C12 myoblasts.

Mitochondria are important targets of oxidative stress in skeletal muscle and participate in redox regulation and cellular antioxidant defense through selenoproteins [19,30]. In this study, SeMet upregulated the mRNA levels of the mitochondrial antioxidant-related selenoprotein genes *Gpx4* and *Txnrd2* (Figure 4A), suggesting that SeMet may enhance mitochondrial selenoprotein-associated antioxidant defense in C2C12 myoblasts under H_2_O_2_-induced oxidative stress. Impaired mitochondrial protein homeostasis can activate MT-UPR, *Gdf15* and *Fgf21* are commonly used as mitochondrial stress-related markers, and their upregulation reflects increased mitochondrial stress [31,32]. In the present study, H_2_O_2_ increased *Gdf15* and *Fgf21* expression and elevated CLPP and JNK2 levels, whereas SeMet attenuated these H_2_O_2_-induced changes (Figure 4A–C). Given the roles of CLPP in mitochondrial protein quality control and JNK2 in stress signaling, these findings suggest that SeMet may mitigate H_2_O_2_-induced mitochondrial stress and attenuate MT-UPR-related pathways in C2C12 myoblasts [19,33].

When proteostasis cannot be restored, prolonged UPR-related cellular stress may promote apoptosis-related pathways [34]. In this study, H_2_O_2_ increased *Bax* and *p53* mRNA expression and elevated BAX protein levels, indicating the activation of pro-apoptotic responses in C2C12 myoblasts (Figure 5A–C). H_2_O_2_ treatment also increased BCL-2 protein expression, which may represent a compensatory anti-apoptotic response to oxidative stress. Compared with the H_2_O_2_ group, SeMet reduced *Bax* and *p53* mRNA expression and decreased both BAX and BCL-2 protein levels, suggesting that SeMet attenuated apoptosis-associated molecular responses that may be linked to unresolved UPR-related cellular stress.

Collectively, this study showed that SeMet treatment alleviated H_2_O_2_-induced oxidative injury in C2C12 myoblasts. This protective effect was associated with increased SELENOF expression, upregulation of mitochondrial selenoprotein-related genes, and attenuation of ER-UPR- and MT-UPR-related responses. Nevertheless, this study was limited to an in vitro C2C12 myoblast model rather than differentiated myotubes or mature skeletal muscle systems. Further validation in differentiated myotubes, primary SMSCs, and animal models is required. In addition, FBS-derived selenium levels were not quantified, which should be addressed in future selenium-defined culture studies.

## 5. Conclusions

SeMet alleviated H_2_O_2_-induced oxidative injury in C2C12 myoblasts. This effect was associated with increased SELENOF expression, attenuation of ER-UPR- and MT-UPR-related responses, and modulation of apoptosis-related markers. These findings provide molecular evidence for further investigating SeMet as a potential regulator of myogenic cell protection and skeletal muscle repair under oxidative stress.

## Figures and Tables

**Table 1:** Gene information and primer sequences used for qRT-PCR analysis.

Gene Symbol	Full Name	Forward Primer Sequence (5′–3′)	Reverse Primer Sequence (5′–3′)
*Selenof*	selenoprotein F	TGGACGACAACGGGAACATTGC	ATGCGTTCCAACTTCTCGCTCAG
*Selenop*	selenoprotein P	GGATGTGGATGGTTGTGAGC	TGGTGATCTGGATCTGGACA
*Gpx4*	glutathione peroxidase 4	CCCGATATGCTGAGTGTGGTTTAC	ATTTCTTGATTACTTCCTGGCTCCTG
*Txnrd2*	thioredoxin reductase 2	CCTCAACCTTAATGGATTACAGCAATG	CACAGCCTCCTCCTCAGACAG
*Gdf15*	growth differentiation factor 15	CTGTTGCTGCTGCTGCTGTC	GCTCGTCGGCGTTGAGTTG
*Fgf21*	fibroblast growth factor 21	ACTGCTGCTGGCTGTCTTCC	TGGTCGTCATCTGTGTAGAGGTAC
*Atf4*	activating transcription factor 4	TCTGCCTTCTCCAGGTGGTTCC	GCTGCTGTCTTGTTTTGCTCCATC
*Xbp1*	X-box binding protein 1	GGTAATGAAGGAGGCCCAGG	CCATGGGAAGGAACTCTATGGC
*Eif2ak3* (*Perk*)	protein kinase RNA-like endoplasmic reticulum kinase	GGTCTTTCTCGGTGGGCATTTCC	CGTCCGCTGAGTCACTGTTGTC
*Hspa5* (*Grp78*)	78 kDa glucose-regulated protein	CCGAGGAGGAGGACAAGAAGGAG	GAACACACCGACGCAGGAATAGG
*Bax*	BCL2-associated X protein	TTCACAGGTTGGCATTAGGAAGGC	ATGGTGAGCGAGGCGGTGAG
*Trp53* (*p53*)	tumor protein p53	ACCGCCGACCTATCCTTACCATC	GGCACAAACACGAACCTCAAAGC
*Gapdh*	glyceraldehyde-3-phosphate dehydrogenase	AGAAGGTGGTGAAGCAGGCATC	CGAAGGTGGAAGAGTGGGAGTTG

## Data Availability

The original contributions presented in this study are included in the article/Supplementary Material. Further inquiries can be directed to the corresponding author(s).

## Supplementary Materials

The following supporting information can be downloaded at: https://www.mdpi.com/article/10.3390/antiox15091099/s1, Table S1: Information of primary antibodies used for western blot analysis.

## Abbreviations

The following abbreviations are used in this manuscript:

Calcein-AM/PI	calcein acetoxymethyl ester/propidium iodide
CCK-8	Cell Counting Kit-8
DMEM	Dulbecco’s modified Eagle’s medium
ER-UPR	endoplasmic reticulum unfolded protein response
FBS	fetal bovine serum
H_2_O_2_	hydrogen peroxide
MT-UPR	mitochondrial unfolded protein response
qRT-PCR	quantitative real-time PCR
ROS	reactive oxygen species
SeMet	selenomethionine
SMSCs	skeletal muscle satellite cells
UPR	unfolded protein response

## References

[1] Sambasivan R., Yao R., Kissenpfennig A., Van Wittenberghe L., Paldi A., Gayraud-Morel B., Guenou H., Malissen B., Tajbakhsh S., Galy A. (2011). Pax7-expressing satellite cells are indispensable for adult skeletal muscle regeneration. Development.

[2] Careccia G., Mangiavini L., Cirillo F. (2024). Regulation of satellite cells functions during skeletal muscle regeneration: A critical step in physiological and pathological conditions. Int. J. Mol. Sci..

[3] Papanikolaou K., Veskoukis A.S., Draganidis D., Baloyiannis I., Deli C.K., Poulios A., Jamurtas A.Z., Fatouros I.G. (2020). Redox-dependent regulation of satellite cells following aseptic muscle trauma: Implications for sports performance and nutrition. Free Radic. Biol. Med..

[4] Ren Y., Li Y., Yan J., Ma M., Zhou D., Xue Z., Zhang Z., Liu H., Yang H., Jia L. (2017). Adiponectin modulates oxidative stress-induced mitophagy and protects C2C12 myoblasts against apoptosis. Sci. Rep..

[5] Duong L.D., West J.D., Morano K.A. (2024). Redox regulation of proteostasis. J. Biol. Chem..

[6] Bhattarai K.R., Riaz T.A., Kim H.R., Chae H.J. (2021). The aftermath of the interplay between the endoplasmic reticulum stress response and redox signaling. Exp. Mol. Med..

[7] Haynes C.M., Ron D. (2010). The mitochondrial UPR—protecting organelle protein homeostasis. J. Cell Sci..

[8] Walter P., Ron D. (2011). The unfolded protein response: From stress pathway to homeostatic regulation. Science.

[9] Hong X., Isern J., Campanario S., Perdiguero E., Ramírez-Pardo I., Segalés J., Hernansanz-Agustín P., Curtabbi A., Deryagin O., Pollán A. (2022). Mitochondrial dynamics maintain muscle stem cell regenerative competence throughout adult life by regulating metabolism and mitophagy. Cell Stem Cell.

[10] Hetz C., Papa F.R. (2018). The Unfolded Protein Response and Cell Fate Control. Mol. Cell.

[11] Xiong G., Hindi S.M., Mann A.K., Gallot Y.S., Bohnert K.R., Cavener D.R., Whittemore S.R., Kumar A. (2017). The PERK arm of the unfolded protein response regulates satellite cell-mediated skeletal muscle regeneration. eLife.

[12] Yildirim A.D., Citir M., Dogan A.E., Veli Z., Yildirim Z., Tufanli O., Traynor-Kaplan A., Schultz C., Erbay E. (2022). ER stress-induced sphingosine-1-phosphate lyase phosphorylation potentiates the mitochondrial unfolded protein response. J. Lipid Res..

[13] Zhang F., Li X., Wei Y. (2023). Selenium and selenoproteins in health. Biomolecules.

[14] Rederstorff M., Krol A., Lescure A. (2006). Understanding the importance of selenium and selenoproteins in muscle function. Cell. Mol. Life Sci..

[15] Lin S., Chen C., Ouyang P., Cai Z., Liu X., Abdurahman A., Peng J., Li Y., Zhang Z., Song G.-L. (2023). SELENOM knockout induces synaptic deficits and cognitive dysfunction by influencing brain glucose metabolism. J. Agric. Food Chem..

[16] Lu C., Qiu F., Zhou H., Peng Y., Hao W., Xu J., Yuan J., Wang S., Qiang B., Xu C. (2006). Identification and characterization of selenoprotein K: An antioxidant in cardiomyocytes. FEBS Lett..

[17] Ren B., Liu M., Ni J., Tian J. (2018). Role of selenoprotein F in protein folding and secretion: Potential involvement in human disease. Nutrients.

[18] Saito Y. (2021). Selenium transport mechanism via selenoprotein P—Its physiological role and related diseases. Front. Nutr..

[19] Jing J., He Y., Liu Y., Tang J., Wang L., Jia G., Liu G., Chen X., Tian G., Cai J. (2023). Selenoproteins synergistically protect porcine skeletal muscle from oxidative damage via relieving mitochondrial dysfunction and endoplasmic reticulum stress. J. Anim. Sci. Biotechnol..

[20] Le Moal E., Pialoux V., Juban G., Groussard C., Zouhal H., Chazaud B., Mounier R. (2017). Redox control of skeletal muscle regeneration. Antioxid. Redox Signal..

[21] Haramizu S., Ota N., Hase T. (2017). Dietary Resveratrol Confers Apoptotic Resistance to Oxidative Stress in Myoblasts. J. Nutr. Biochem..

[22] Liu Y., He A., Tang J., Shah A.M., Jia G., Liu G., Tian G., Chen X., Cai J., Kang B. (2021). Selenium alleviates the negative effect of heat stress on myogenic differentiation of C2C12 cells with the response of selenogenome. J. Therm. Biol..

[23] Schrauzer G.N. (2000). Selenomethionine: A review of its nutritional significance, metabolism and toxicity. J. Nutr..

[24] Burk R.F., Hill K.E. (2015). Regulation of selenium metabolism and transport. Annu. Rev. Nutr..

[25] Suzuki K.T. (2005). Metabolomics of selenium: Se metabolites based on speciation studies. J. Health Sci..

[26] Minich W.B. (2022). Selenium metabolism and biosynthesis of selenoproteins in the human body. Biochemistry.

[27] Jing J., Yin S., Liu Y., Liu Y., Wang L., Tang J., Jia G., Liu G., Tian G., Chen X. (2022). Hydroxy selenomethionine alleviates hepatic lipid metabolism disorder of pigs induced by dietary oxidative stress via relieving the endoplasmic reticulum stress. Antioxidants.

[28] Chen X., Shi C., He M., Xiong S., Xia X. (2023). Endoplasmic reticulum stress: Molecular mechanism and therapeutic targets. Signal Transduct. Target. Ther..

[29] Lebeau J., Saunders J.M., Moraes V.W.R., Madhavan A., Madrazo N., Anthony M.C., Wiseman R.L. (2018). The PERK arm of the unfolded protein response regulates mitochondrial morphology during acute endoplasmic reticulum stress. Cell Rep..

[30] Chen M.M., Li Y., Deng S.L., Zhao Y., Lian Z.X., Yu K. (2022). Mitochondrial function and reactive oxygen/nitrogen species in skeletal muscle. Front. Cell Dev. Biol..

[31] Forsström S., Jackson C.B., Carroll C.J., Kuronen M., Pirinen E., Pradhan S., Marmyleva A., Auranen M., Kleine I.-M., Khan N.A. (2019). Fibroblast growth factor 21 drives dynamics of local and systemic stress responses in mitochondrial myopathy with mtDNA deletions. Cell Metab..

[32] Jena J., García-Peña L.M., Pereira R.O. (2023). The roles of FGF21 and GDF15 in mediating the mitochondrial integrated stress response. Front. Endocrinol..

[33] Romanelli S., Trempe J.F. (2025). Stressful situations: Molecular insights on mitochondrial quality control pathways. J. Biol. Chem..

[34] Chen X., Yin X.M. (2011). Coordination of autophagy and the proteasome in resolving endoplasmic reticulum stress. Vet. Pathol..

