# Supplementary material for: Selenomethionine Attenuates Hydrogen Peroxide-Induced Oxidative Injury and Modulates Unfolded Protein Response-Related Markers in Murine C2C12 Myoblasts
Source: Antioxidants (Basel). 2026 Aug 31;15(9):1099. doi: 10.3390/antiox15091099 (PMC13603669; doi:10.3390/antiox15091099)
Supplement: Supplementary file 1 [file antioxidants-15-01099-s001.zip › antioxidants-4476326-supplementary.pdf]

Table S1. Information of primary antibodies used for western blot analysis.

Target	Manufacturer	Catalog No.	Clone	Host	Dilution
SELENOP	Bioss	bsm-50198M	37A1	Mouse	1:1000
SELENOF	Abcam	ab124840	NCIR128A	Rabbit	1:1000
GRP78	Bioss	bsm-51623M	D12H2	Mouse	1:1000
XBP1	Bioss	bs-23974R	-	Rabbit	1:1000
CLPP	Bioss	bsm-62275R	12G6	Rabbit	1:1000
JNK2	Bioss	bs-2592R	-	Rabbit	1:1000
BAX	Bioss	bsm-52316R	3C3	Rabbit	1:1000
BCL-2	Bioss	bsm-61074R	B9E6	Rabbit	1:1000
β-actin	Bioss	bsm-63325R	2B3	Rabbit	1:1000
